# Multidimensional poverty of persons with disabilities in China: An analysis of poverty reduction effect of employment services

**DOI:** 10.3389/fpubh.2023.1093978

**Published:** 2023-02-10

**Authors:** Xiaofeng Wang, Jiamin Guo, Hu Li

**Affiliations:** Northeast Asian Research Center, Jilin University, Changchun, China

**Keywords:** disability, multidimensional poverty, employment services, AF method, PSM-DID

## Abstract

**Introduction:**

Disability is a global public health problem, and poverty due to illness and disability has always been a major problem and challenge for global poverty governance. In order to eradicate poverty, China has carried out a series of welfare reforms and employment interventions for people with disabilities. The purpose of this study is to examine the levels of multidimensional poverty of persons with disabilities aged 16–59 in China and the poverty reduction effect of employment services.

**Methods:**

The Alkire-Foster (AF) method is applied to measure and decompose the multidimensional poverty index (MPI) of people with disabilities in this study. In order to make the results more robust, ordinary least squares (OLS) regression and the combination of propensity score matching and difference-in-differences (PSM-DID) are used to study the effect of employment services on multidimensional poverty of the disabled.

**Results:**

The results show that among persons with disabilities aged 16–59, about 90% are deprived in at least one dimension, and about 30% are in a state of severe multidimensional poverty until 2019. The contributions of deprivation in the dimensions of education and social participation are remarkably higher than dimensions of economy, health and insurance. In addition, employment services have a significant improvement effect on multidimensional poverty, which is reflected not only in the economic dimension, but also in the dimensions of education, insurance and social participation.

**Conclusion:**

People with disabilities are generally in multidimensional poverty in China, and their abilities in learning and social integration are seriously inadequate. Employment services have played a great role in improving poverty, but the improvement has been different in different dimensions and different disability categories. These findings provide important evidence for recognizing multidimensional poverty of persons with disabilities and the poverty reduction effect of employment services, which will help to formulate more reasonable public policies to eradicate poverty.

## 1. Introduction

Disability is a complex situation which affects individuals and families around the world. According to the World Health Organization (WHO) and the World Bank (WB), about 15% of the world's population lives with some form of disability, and 80% of those with disabilities live in low- and middle-income countries (1). According to the data of China's sixth census and the Second China National Sample Survey on Disability, the total number of persons with disabilities in China at the end of 2010 was 85.02 million (2). Poverty due to illness and disability is a long-term and complex problem, which has always been a major problem and challenge for global poverty governance. China has a large population with disabilities, and poverty due to disability is common. In 2018, the poverty rate due to disability has exceeded 14% (3). By the end of 2020, China's poverty alleviation goals and tasks have been fully completed as scheduled, but the deprivation of education, health, housing and other benefits has become the main manifestation of poverty. In the post poverty alleviation era, the poverty alleviation goal will face a strategic transformation, that is, from absolute poverty to relative poverty. Due to factors such as physical disabilities, limited labor capacity, and low educational attainment, persons with disabilities are more likely to suffer from poor quality of life and become a prominent group in relative poverty (1, 4). Therefore, in the process of consolidating the achievements of poverty alleviation, it is necessary not only to prevent the disabled from returning to poverty, but also to help them improve their health, education, social security, etc., that is, on the basis of solving the absolute poverty of the disabled, it is necessary to improve the multidimensional poverty with insufficient basic capabilities.

The analysis of poverty has gone beyond the lack of income and material, and entered into the study of the nature of poverty from the dimensions of human development and spirituality. The capability theory of Sen has been at the forefront of the movement away from the unidimensional income approach to poverty measurement, with Sen defining poverty as a lack of freedom due to the deprivation of basic capability (5). The capability not only has the tool value of eliminating poverty, but also represents a kind of human welfare. Based on this theory, relevant studies have shown that in most developing countries, disability is found to be significantly associated with higher multidimensional poverty as well as lower educational attainment, lower employment rates, and higher medical expenditures (6). Persons with disabilities have a lower quality of life and face a higher risk of multidimensional poverty (7), and social policies should aim to reduce their high levels of multidimensional poverty and deprivation of capabilities (8).

Based on the severe multidimensional poverty of the disabled, relevant studies have pointed out that employment can be an effective way to improve personal wellbeing (9), and full and effective employment is a key link to realize the replacement of “blood transfusion” assistance with “hematopoietic” support and the replacement of maintaining basic survival guarantee with promoting development-oriented welfare in the field of disabled people's undertakings. However, persons with disabilities consistently have lower employment rates than non-disabled persons (10), and face significant challenges in wages, promotion, workplace and accommodation (11). The employment of persons with disabilities has become a long-term concern of many governments around the world, and has led to a series of welfare reforms and employment intervention policies. But it appears that putting the actual policies and legislation into practice has had limited success across China and, for the most part, China's disabled population remains out of the mainstream wherein access to education, training, and good-paying jobs are a minimum (12). Long-term unemployment for persons with disabilities is serious problem in China, with millions of unemployed disabled persons living in poverty (13). Therefore, reasonable and effective employment support measures are the key to solve the multidimensional poverty problem.

Employment services play an important role in promoting and ensuring the employment of persons with disabilities. In 2007, China promulgated the Regulations on Employment of Persons with Disabilities (14). Employment services are an important part of the regulations, and it is very necessary to evaluate the current implementation effect. As an important intervention means to alleviate poverty and improve the quality of life of the disabled, employment services have been evaluated by some scholars. The effectiveness of different occupational interventions in improving employment, educational, clinical, and quality-of-life outcomes for persons with psychiatric disabilities has been evaluated, and the research shows that the vocational and educational outcomes have been significantly improved (15). Some studies have pointed out that vocational rehabilitation services aimed to improve life functioning will lead to an improvement in quality of life (16, 17). The Individual Placement and Support (IPS) has been studied by several scholars and has shown that it can increase life satisfaction and time spent in daily occupations and community life for persons with disabilities (18), and it is effective in gaining employment and integrating into the local community (19). It can be seen that the role of employment services on disabled people may not only be reflected in the economy. It is more thorough and comprehensive to evaluate the poverty reduction effect of employment services from the perspective of multidimensional poverty of the disabled.

**Figure 1:**
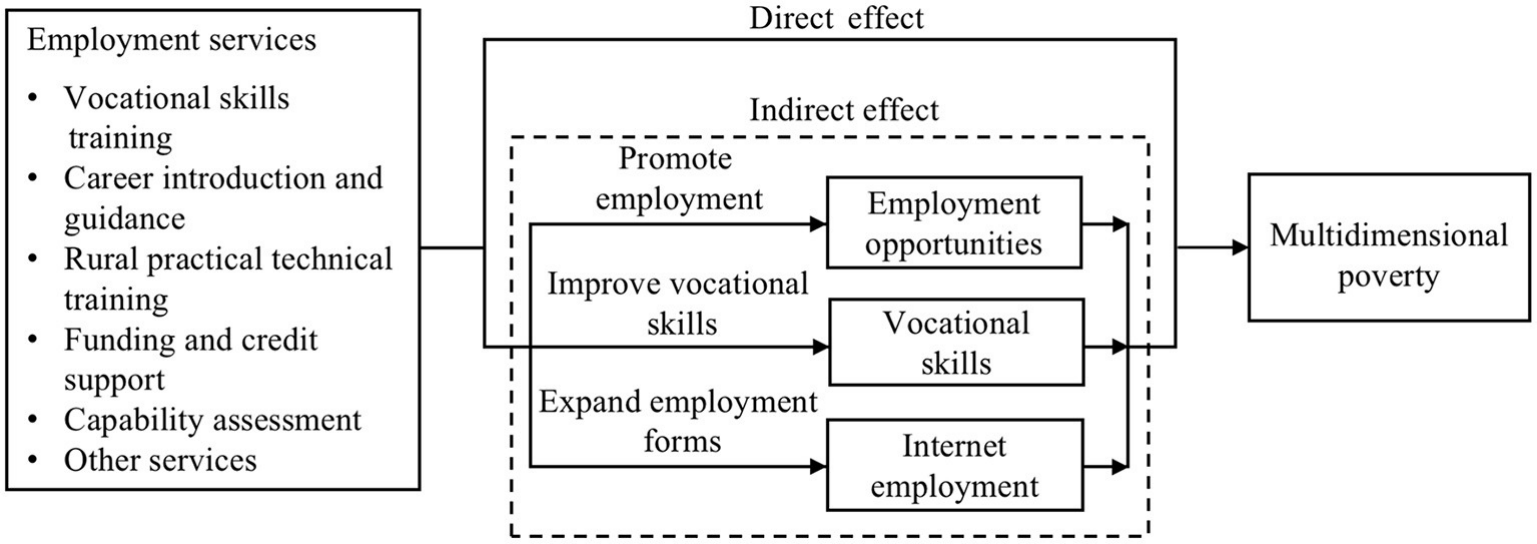
The mechanism of employment services to improve multidimensional poverty of persons with disabilities.

The goal of the employment service is to promote persons with disabilities to be in a state of optimal economic self-sufficiency, and the ultimate goal is that the recipient of employment services should experience improvement in his or her quality of life. Therefore, the evaluation of the poverty reduction effect of employment services should not be limited to income, but should focus on the perspective of multidimensional poverty. In addition, achievement of the ultimate goal can be expected to result from a process in which vocational outcomes such as the improvement of employment opportunities, vocational skills and employment forms are obtained *via* targeted employment services. In view of the above analysis, the data in Basic Service Status and Demand Information for Persons with Disabilities registered in 2018 and 2019 were used in this study. First, this paper expands the poverty measurement standard of the disabled from one-dimensional income poverty to multidimensional capabilities poverty, and identifies multidimensional poverty from five dimensions of economic capacity, physical function, learning capacity, risk resistance and social integration capacity. Second, the improvement effect of employment services on multidimensional poverty of the disabled is evaluated from a micro perspective. Finally, in order to gain a deeper understanding of the relationship between employment services and multidimensional poverty of the disabled, the mediation effects of the three vocational outcomes of employment opportunities, vocational skills and Internet employment are verified. Based on the above analysis, this paper proposes the mechanism of employment services to improve multidimensional poverty of persons with disabilities (as shown in Figure 1).

Based on the collation and analysis of the literature, we can find that there are still some deficiencies in the current research. First, China has achieved remarkable results in poverty reduction. However, it cannot be ignored the fact that exclusion of disabled persons from education, employment, social and political activities have not been improved by the increase in their income (20). A single monetary indicator does not capture other negative aspects that affect persons with disabilities and their families, such as social exclusion. Therefore, it is fundamental to analyze the poverty situation of the disabled from a multidimensional perspective. However, the existing researches on multidimensional poverty mostly focus on rural households, and there are relatively few studies on the multidimensional poverty of the disabled, especially in China. Second, the existing research focuses on theoretical analysis and evaluation of employment services for the disabled, lacking empirical data. A review of the studies suggests that China's employment legislation is quite progressive in terms of access to employment and in terms of protecting employment rights of persons with disabilities, but there appears to be a huge disconnection between the actual legislative policies and implementation of the stated policies (12, 20). Social services, rehabilitation services, employment services and other services are insufficient to meet development needs, resulting in poverty for the majority of persons with disabilities (20). A scholar explained qualitatively why the actual employment situation in China stagnated or even worsened despite the impression that employment support for the disabled was improving (21). It can be seen that most scholars believe that the actual effect of employment services is extremely limited in China. It is worth noting that these studies are mostly macro qualitative analysis, lacking quantitative demonstration using micro data. Third, some scholars pointed out that employment should not be used as the sole indicator of rehabilitation success. They advocate that assessing changes attributable to vocational interventions should focus on the impact of improvement of life skills through vocational interventions on multiple areas of quality of life (17). A number of scholars have evaluated the effects of employment services for persons with disabilities in terms of employment, education, daily activities, community integration, quality of life, etc. (15–18). However, there are still shortcomings in the current research. On the one hand, most of the research only takes the mental disabled as the research object, which leads us to not have a more comprehensive understanding of the disabled group. On the other hand, most studies only evaluate employment support measures from a single area of the living conditions of the disabled, which may underestimate the impact of employment support measures on the disabled.

Based on the above analysis, this paper has the following contributions to measure multidimensional poverty of the disabled and to evaluate the poverty reduction effect of employment services. First, this paper builds a multidimensional poverty index system for the disabled based on Sen's capability theory, which not only enriches the research results of the application of capability theory to the field of the disabled in China, but also helps to improve our overall understanding of the poverty situation of the disabled. Second, based on the existing qualitative research on the employment support policies for the disabled in China, quantitative analysis using micro data can provide more accurate evidence for the implementation effect of employment support policies. Third, employment service is a development-oriented policy aimed at disabled persons with working ability in China's poverty alleviation policy. Multidimensional poverty involves multiple areas of disabled persons' lives. This paper evaluates employment services and analyzes different categories of disabled people from the perspective of multidimensional poverty, which makes the evaluation of employment service more comprehensive and accurate. In addition, this study provides guidance for China to alleviate poverty by improving the employment service system in the post-poverty era. Fourth, the PSM-DID method of causal inference is used for analysis, which helps to solve the endogenous problem in the model and ensures the reliability of the benchmark regression results.

## 2. Materials and methods

### 2.1. Data

The data used in this study come from Basic Service Status and Demand Information for Persons with Disabilities. This is a large-scale survey organized and coordinated by the State Council Disabled Persons' Working Committee, and the specific work is undertaken by the China Disabled Persons' Federation. The purpose is to grasp the basic service status and needs of persons with disabilities in a timely manner, establish and improve the national basic database of certified persons with disabilities, and promote the high-quality development of the cause of persons with disabilities. The subjects of the investigation are the disabled who hold the Disabled Person Certificate of the People's Republic of China. The survey content includes main survival and development items (basic information, housing, education, employment, social security, basic medical care and rehabilitation, accessibility, culture and sports, etc.) and basic public service facilities in all villages (communities) across the country. Due to the difficulty in obtaining data, this paper only uses the data of Jilin Province in 2018 and 2019 for research, and takes the working-age disabled people aged 16–59 as the main research group. Finally, the 435,312 samples in 2018 are selected and the information in 2019 is tracked.

### 2.2. Analysis method: Multidimensional poverty measurement

#### 2.2.1. Alkire-Foster method

Alkire and Foster, scholars of Oxford Poverty and Human Development Initiative (OPHI), proposed AF method to measure multidimensional poverty (22). At present, the AF method has become the mainstream method to measure multidimensional poverty and has been recognized by the United Nations Development Progarmme (UNDP). The AF method was adopted in our study to measure multidimensional poverty based on the capability theory. The fundamental logic of selecting the AF method is that it admits the independence of several dimensions of poverty, and it also highlights the threshold of the number of deprived dimensions to be multidimensional poverty. Generally, three key processes were used to accomplish the measurement: identification, aggregation and decomposition. For the process of identification, the method of dual cutoff identification is adopted to identify poor disabled persons according to two distinct cutoffs: deprivation and poverty cutoffs. First, a deprivation cutoff is used to identify whether a person with disabilities is deprived in each dimension, and each disabled person's deprivation score is defined as the sum of weighted scores of all dimensions. Second, a poverty cutoff is used to determine whether a person with disabilities could be considered multidimensionally poor. A disabled person is identified as multidimensionally poor if his or her deprivation score is greater than or equal to a given poverty cutoff (k) (22, 23).

The process of aggregation needs to be imitated to aggregate multiple dimensions to get a composite index after identifying the deprivation of dimensions of poverty. The aggregation step of our methodology proposes an adjusted headcount ratio, which builds upon the standard FGT technology and overcomes its shortcomings (22). The adjusted headcount ratio (*M*_0_), also referred to the MPI, is calculated by multiplying the incidence (*H*) and intensity (*A*) of poverty. The incidence of poverty (*H*), namely the multidimensional headcount ratio, is defined as the proportion of persons with disabilities who are poor in multiple dimensions. It is calculated as the number of persons with disabilities identified as multidimensionally poor (*q*) divided by the total number of persons with disabilities (*n*). The intensity of poverty (*A*) indicates the average deprivation score among the poor. Based on the above description, *M*_0_ is defined as follows:


(1) $$\begin{array}{l} M_{0}=H\times A=\frac{q}{n}\times \frac{\sum_{\;i=1}^{n}c_{i}\left(k\right)}{qd}=\frac{\sum_{\;i=1}^{n}c_{i}\left(k\right)}{nd} \end{array}$$

where *c*_*i*_(*k*)/*d* represents the share of possible deprivations experienced by a poor person *i*, and hence the average deprivation share across the poor is given by $A=\overset{n}{\underset{\;i=1}{\sum }}c_{i}\left(k\right)/\left(qd\right).$ This partial index conveys relevant information about multidimensional poverty, namely, the fraction of possible dimensions *d* in which the average poor person is deprived.

Decomposition analysis can provide targeted policies for poverty alleviation. In this study, *M*_0_ was decomposed according to dimensions in order to reveal the underlying structure of deprivation. Dimensional breakdown refers to the contribution of each deprivation dimension to overall poverty (*M*_0_). Therefore, the contribution of each dimension (*P*_0*j*_) is calculated as the poverty index for each dimension (*M*_0*j*_) divided by the overall poverty index (*M*_0_).

#### 2.2.2. Selecting dimensions, indicators and deprivation cutoff

Measurement of poverty is an essential prerequisite for targeting interventions and designing programs and policies to alleviate poverty. Given its multidimensional nature, there are various indicators, such as income, expenditure, deprivation, and social exclusion, that could be used to measure multidimensional poverty (24). UNDP has established a multidimensional human development index to measure the development level of each country, including three dimensions of education, health and living standards (25), which has been adopted by the existing studies of multidimensional poverty (8, 26–28). Relevant research scholars have expanded the measurement dimensions of multidimensional poverty. Four dimensions of health (disability), education, housing and living standards were used to measure multidimensional poverty among Iranian older adults (29); six dimensions of economy, health, housing, employment, social protection and interpersonal relationship were used to measure multidimensional poverty of disabled families in South Korea (30); other dimensions are added to measure multidimensional poverty of persons with disabilities in different countries, such as social participation, information and communication, basic daily activities, subjective and psychological wellbeing, etc. (31, 32). It can be seen that there is no consensus on how to design a multidimensional poverty measure, and the most appropriate deprivation indicators and the results of multidimensional poverty studies vary across countries, regions, and groups.

In this research, five deprived dimensions—economy, health, education, insurance and social participation—including eight indicators were selected to form our MPI based on previous studies and the availability of data. According to the construction method issued by UNDP, each dimension is assigned equal weight (the indicators under each dimension are also assigned equal weight) to reflect the equal importance of each dimension in the evaluation system (25). The two indicators of income and housing are used to measure the dimension of economy. The poverty line in income is not fixed but changes along with economic development. In the process of registering disabled information, the income of disabled people will be divided into different categories according to the poverty line of the year. Previous studies only used income to reflect economic status (30), and housing situation was added to this paper to deepen the study of economic capacity. As far as health dimension is concerned, a series of variables, such as nutrition, child mortality, self-assessed health status, and the presence of a chronic illness are usually selected to measure the health deprivation (8, 23). Due to the limitation of registration content, the indicator of other diseases besides disability is used to measure the physical function of persons with disabilities, and the health dimension needs to be further improved. As far as education dimension is concerned, two indicators are often selected: years of schooling (primary school incomplete for adults) and child school attendance (children aged 6–16 years old are not attending to school) (8, 33). Persons with disabilities aged 16–56 are the research group in this paper, and educational attainment plays a crucial role in their lives, so adults' completion of primary school is used as an indicator to measure the educational dimension. As for insurance dimension, health insurance is selected to measure the insurance deprivation (30). For persons with disabilities, the capacity to resist risk is critical, and medical insurance and pension insurance are chosen to represent insurance status. As for social participation dimension is concerned, participation in community activities is usually selected to measure its deprivation (31), but such measurement is somewhat singular. This paper tries to evaluate social participation from multiple perspectives. The Internet is an important channel for information acquisition and communication, especially for the working-age population, its role in social life is indispensable. Thus, participation in social activities and the use of Internet are chosen to represent social participation status.

Table 1 presents the deprivation dimensions, indicators, and cutoffs used in this study. The levels of multidimensional poverty vary according to the selection of poverty cutoffs (k). Based on previous studies, a cutoff (k = 0.33) is selected because it has a normative justification and provided a wide distribution of poverty results (25). That is, a person is considered multidimensionally poor if his or her weighted sum of deprivations is equal to 33% or higher. In addition to this multidimensional poverty line, severe (50%) and vulnerable (20%) poverty cutoffs are used to calculate the percentage of people who are severely multidimensionally poor and vulnerable to multidimensional poverty (34). In order to make the results more objective, variable poverty cutoffs (20–50%) are adopted in this section to describe the levels of multidimensional poverty for persons with disabilities.

**Table 1:** Dimensions, indicators, deprivation criteria and weights.

**Dimension**	**Indicator**	**Deprivation cutoff (deprived if …)**	**Weight**
Economy	Income level	The person's average annual income is below the national poverty line	1/10
	Housing situation	The person has no housing title in urban areas or poor housing conditions in rural areas	1/10
Health	Other diseases besides disability	The person has other diseases besides disability	1/5
Education	Education level	The person has not completed primary school education	1/5
Insurance	Medical insurance	The person has no medical insurance	1/10
	Pension insurance	The person has no pension insurance	1/10
Social participation	Cultural and sports activities	The person does not often participate in cultural and sports activities	1/10
	Internet	The person does not use the internet	1/10

### 2.3. Analysis method: The poverty reduction effect of employment services

#### 2.3.1. Benchmark model

In order to preliminarily investigate the impact of employment services on the MPI of the disabled, ordinary least squares (OLS) is used in this paper, and the model is set as follows:


(2) $$\begin{array}{l} M_{i}=\beta_{0}+\beta_{1}S_{i}+\beta_{j}X_{i}{+\varepsilon }_{i} \end{array}$$

where *i* represents the individual with disabilities; *M*_*i*_ is the explained variable, representing the MPI of the disabled; *S*_*i*_ is the explanatory variable, representing that the disabled are provided with employment services; *X*_*i*_ represents a series of covariates such as personal, family and social support of the disabled; ε_*i*_ represents random error term. The explained variable is a categorical variable, so OLS is selected for regression analysis.

#### 2.3.2. PSM-DID

It is worth noting that persons with disabilities in severe multidimensional poverty are more likely to receive employment services, social assistance, welfare subsidies and other assistance, that is, whether persons with disabilities receive employment services is not random, but is affected by their own characteristics and needs. Therefore, in order to solve endogenous problems such as sample selection bias and missing variables, the PSM-DID method is adopted to investigate the improvement effect of employment services on multidimensional poverty.

The DID method has advantages in investigating the effectiveness of policy by comparing the differences in the effects of a certain policy between the treatment group and the control group. In the DID model, the treatment group and the control group must meet the parallel trend assumption in the base period, however, in reality, the parallel trend assumption cannot be easily satisfied (35). In order to solve this problem, this study introduces the PSM method to match the treatment group and the control group in the base period. By controlling the heterogeneity of the two groups, the treatment group and the control group after the base period matching are closer to the natural experiment, so as to ensure that the DID method meets the parallel trend assumption to a certain extent. The PSM-DID regression approach can be applied for causal inferences to counter selection bias or confounding (36), and is widely used in academic research (35, 37–40).

We used the samples after conducting a panel balance for the data in 2018 and 2019. The samples that did not receive employment services in 2018 and 2019 were defined as control group *C*, and the samples that did not receive employment services in 2018 but received employment services in 2019 were defined as treatment group *T*. At the same time, age, gender, hukou status, disability categories, disability grades, marital status and other welfare policies were selected as the control variables. The fixed effect model constructed in this paper is as follows:


(3) $$\begin{array}{l} M_{it}=\beta_{2}+\beta \;treat_{i}{\times time}_{t}+\rho X_{it}{+{\mu_{i}+\vartheta }_{t}+\varepsilon }_{it} \end{array}$$

where *M*_*it*_ is the explained variable, which represents the MPI of the disabled, *i* represents the individual with disabilities, and *t* represents the time before and after the disabled obtain employment services; the treatment variable *treat*_*i*_ is a binary indicator that represents the group dummy variable, *treat* = 1 represents the treatment group that the individuals obtain employment services, and *treat* = 0 represents the control group that the individuals do not obtain employment services; *time*_*t*_ represents the time dummy variable, *time* = 1 represents the time after the implementation of employment services (the year of 2019), and *time* = 0 represents the time before the implementation of employment services (the year of 2018); the variable *treat*_*i*_×*time*_*t*_ denotes the interaction between groups and time; *X*_*it*_ represents a set of individual covariates of disabled *i* at time *t*; μ_*i*_ and ϑ_*t*_ represent the individual and time level fixed effects, respectively; ε_*it*_ denotes the random error term and contains information other than the main variables of the model. β is calculated by the PSM-DID model and represents the net effect of employment services on the improvement of multidimensional poverty. The formula for calculating β is as follows:


(4) $$\begin{array}{l} \beta =E\;(Y_{2019i}^{T}-Y_{2018i}^{T}\;|\;p(X_{2018i}),D_{i}=1)\\ \;-E\;(Y_{2019i}^{C}-Y_{2018i}^{C}\;|\;p(X_{2018i}),D_{i}=0) \end{array}$$

where *Y*_2018*i*_ represents the MPI of individuals in 2018, and *Y*_2019*i*_ represents the MPI of individuals in 2019; *p*(*X*_2018*i*_) is the probability of obtaining employment services in 2019 calculated according to the characteristics of individuals in 2018. *D*_*i*_ represents the treatment variable in 2018, *D*_*i*_ = 1 represents the treatment group, and *D*_*i*_ = 0 represents the control group.

#### 2.3.3. Mediation effect model

In the above mechanism analysis, we propose that employment services may improve multidimensional poverty of the disabled by promoting employment, improving employment skills and promoting internet employment. In order to test the mediation effect, the model is constructed as follows:


(5) $$\begin{array}{l} M_{it}=\beta_{0}+\beta_{1}S_{it}+\beta_{j}X_{it}{{+\mu_{i}+\vartheta }_{t}+\varepsilon }_{it} \end{array}$$

(6) $$\begin{array}{l} Z_{it}=\alpha_{0}+\alpha S_{it}+\beta_{j}X_{it}{+{\mu_{i}+\vartheta }_{t}+\varepsilon }_{it} \end{array}$$

(7) $$\begin{array}{l} M_{it}=\theta_{0}+\theta S_{it}+\theta_{1}Z_{it}+\beta_{j}X_{it}{+\mu_{i}+\vartheta_{t}+\varepsilon }_{it} \end{array}$$

In formula (5), the coefficient β represents the total effect of employment services on the MPI. In formula (6), the coefficient α represents the effect of employment services on the mediation variable *Z*_*it*_. In formula (7), the coefficient θ represents the direct effect of employment services on the MPI under the control of the mediation variable, and the coefficient θ_1_ represents the effect of the mediation variable on the MPI under the control of employment services. When β is significant in formula (5), the mediation effect is jointly tested by formulas (6) and (7). If α, θ and θ_1_ are all significant, there is a partial mediation effect; if α and θ_1_ are significant, θ is not significant, there is a full mediation effect.

## 3. Results

### 3.1. Multidimensional poverty estimates

Table 2 presents *H*, *A* and *M*_0_ for persons with disabilities aged 16–59 under different poverty cutoffs. First, the incidence of poverty (*H*) in 2018 varies from 93.9% (k = 20%) to 78.0% (k = 30%) and 39.0% (k = 50%). Meanwhile, *M*_0_ values are 0.390, 0.358, and 0.220 for these three cutoffs, respectively. With the increase of the poverty cutoff, *H* and *M*_0_ show a downward trend, and the same trend is shown in 2019. This is because the increase of the poverty cutoff means that the threshold of identifying multidimensional poverty rises, and the number of people who reach the threshold of multidimensional poverty decreases. Second, with the increase of the poverty cutoff, *A* shows an increasing trend. Since *A* reflects the poverty intensity of the poor, the disabled who are in poverty are deprived in more dimensions when the multidimensional poverty threshold rises, which leads to an increase in the poverty intensity. Third, compared with 2018, *H*, *A*, and *M*_0_ all decreased to varying degrees in 2019, which shows that the levels of multidimensional poverty among persons with disabilities has decreased. In general, the results show that about 90% are deprived in at least one dimension, and about 30% are in a state of severe multidimensional poverty until 2019. It can be seen that the disabled faced a higher level of multidimensional poverty.

**Table 2:** Incidence (*H*), Intensity (*A*), and MPI (*M*_0_) for different poverty cutoffs.

**Cutoffs (k)**	**Year**	**Incidence (H)**	**Intensity (A)**	**MPI (M_0_)**
0.2	2018	93.9%	41.5%	0.390
	2019	90.8%	39.2%	0.356
0.3	2018	78.0%	45.9%	0.358
	2019	71.9%	44.2%	0.318
0.4	2018	60.3%	50.6%	0.305
	2019	53.5%	49.2%	0.263
0.5	2018	39.0%	56.4%	0.220
	2019	31.7%	55.5%	0.176

In order to see the deprivation degree of the disabled in each dimension, we decompose the MPI into the sum of the poverty index of each dimension, and then calculate the percentage contribution. Table 3 presents *M*_0*j*_ and *P*_0*j*_ for persons with disabilities aged 16–59. Under different poverty cutoffs, *P*_01_, *P*_02_ and *P*_04_ change slightly, and are basically below 20%, indicating that the dimensions of economy, health and insurance present similar levels of deprivation. Compared with 2018, *P*_01_, *P*_02_ and *P*_04_ decrease in 2019, indicating that poverty in the dimensions of economy, health and insurance has improved. The year 2020 is the year for China to achieve the goal of building a moderately prosperous society in all respects, and the end of the battle to win the fight against poverty in all respects. Toward 2020, China's poverty reduction policy is committed to solving absolute poverty and improving people's livelihood. The dimensions of economy, health and insurance are directly related to the basic survival of persons with disabilities, which has been greatly improved. In addition, *P*_03_ and *P*_05_ are higher than 20%, indicating that the disabled have higher levels of deprivation in the dimensions of education and social participation. The disabled face many difficulties in education and social participation due to physiological defects. It is necessary to further improve the education system for the disabled and strengthen the infrastructure and information construction.

**Table 3:** Decomposition results of MPI for different poverty cutoffs: different dimensions.

**k**	**Year**	**Economy**		**Health**		**Education**		**Insurance**		**Social participation**	
		**M**_**01**_	**P**_**01**_	**M**_**02**_	**P**_**02**_	**M**_**03**_	**P**_**03**_	**M**_**04**_	**P**_**04**_	**M**_**05**_	**P**_**05**_
0.2	2018	0.074	18.9%	0.007	1.8%	0.081	20.7%	0.057	14.6%	0.172	44.0%
	2019	0.066	18.6%	0.005	1.4%	0.075	21.1%	0.047	13.2%	0.163	45.7%
0.3	2018	0.071	19.9%	0.007	1.9%	0.081	22.5%	0.054	15.1%	0.145	40.6%
	2019	0.063	19.8%	0.005	1.5%	0.075	23.5%	0.043	13.6%	0.132	41.5%
0.4	2018	0.061	20.0%	0.006	2.1%	0.078	25.7%	0.045	14.6%	0.115	37.6%
	2019	0.052	19.8%	0.004	1.7%	0.071	27.1%	0.034	12.9%	0.101	38.5%
0.5	2018	0.045	20.5%	0.006	2.5%	0.061	27.8%	0.032	14.6%	0.076	34.6%
	2019	0.037	21.0%	0.004	2.1%	0.050	28.3%	0.024	13.4%	0.062	35.1%

There are large internal differences among persons with disabilities. In order to see the deprivation degree of the disabled in different disability categories, we decompose the MPI into the sum of the poverty index of each disability category. Table 4 shows *M*_0*j*_ of persons with different disability categories aged 16–59. Under different poverty cutoffs, the multidimensional poverty of persons with different disability categories presents the same characteristics, that is, *M*_0*j*_ of persons with intellectual disabilities is the highest, and *M*_0*j*_ of persons with physical disabilities is the lowest. It can be seen that persons with intellectual, multidimensional, psychiatric or speech disabilities face a serious poverty situation, while those with physical, visual or hearing disabilities have a relatively good living condition. From the perspective of time, *M*_0*j*_ of all categories of disabled persons in 2019 has decreased compared with that in 2018, which indicates that the living conditions of all categories of disabled people have improved, and the improvement of persons with physical, hearing or speech disabilities is relatively large. In the process of poverty reduction, the diversity within the disabled deserves attention.

**Table 4:** Decomposition results of MPI for different poverty cutoffs: different disability categories.

**Cutoffs (k)**	**Year**	**Visual disability**	**Hearing disability**	**Speech disability**	**Physical disability**	**Intellectual disability**	**Psychiatric disability**	**Multiple disability**
0.2	2018	0.369	0.368	0.426	0.355	0.509	0.434	0.459
	2019	0.338	0.329	0.388	0.316	0.485	0.409	0.434
0.3	2018	0.332	0.332	0.404	0.315	0.500	0.413	0.442
	2019	0.295	0.287	0.361	0.269	0.475	0.384	0.414
0.4	2018	0.274	0.276	0.360	0.254	0.478	0.363	0.406
	2019	0.235	0.227	0.315	0.208	0.449	0.329	0.375
0.5	2018	0.187	0.186	0.275	0.168	0.411	0.271	0.324
	2019	0.147	0.138	0.213	0.124	0.366	0.230	0.280

### 3.2. The poverty reduction effect of employment services

#### 3.2.1. Descriptive statistics of variables

Explained variable: MPI. According to relevant literature (26, 34), k = 0.33 is set as the poverty cutoff in this part. Persons with disabilities whose MPI is ≥ 0.33 are considered to be in multidimensional poverty, and the higher the MPI, the more severe multidimensional poverty of the disabled.

Explanatory variable: employment service. Employment services in Regulations on the Employment of Persons with Disabilities are mainly provided free of charge by the China Disabled Persons' Federation and its affiliated employment service agencies for the disabled. In the registration form of Basic Service Status and Demand Information for Persons with Disabilities formulated by China Disabled Persons' Federation, employment services include vocational skills training, career introduction, rural practical technical training, funding and credit support, vocational guidance, etc. The employment service is set as a binary variable, which is assigned a value of 1 if the disabled person obtains any of the above services, otherwise, it is assigned a value of 0.

Control variable: individual characteristics, family characteristics and social support. Individual characteristics include gender, age, hukou status, disability level, and disability type. Among them, gender and hukou status are both binary variables; disability categories (multiple, visual, hearing, speech, physical, intellectual and psychiatric disabilities) and disability grades (grades 1–4) are categorical variables. Family characteristics include marital status, which is assigned a value of 1 if the disabled person has a spouse, otherwise, it is assigned a value of 0. Social support includes social assistance, welfare subsidies, rehabilitation services and barrier-free reconstruction, all of which are binary variables.

Mediation variable: employment opportunity is a binary variable, which is assigned a value of 1 if the disabled person is employed, otherwise, it is assigned a value of 0; vocational skill is set as a categorical variable, representing the number of vocational skills mastered by persons with disabilities; Internet employment is a binary variable, which is assigned a value of 1 if the disabled person uses the Internet for employment, otherwise, it is assigned a value of 0. Table 5 shows the specific definitions of each variable.

**Table 5:** Definition of variables.

**Variable**	**Variable type**	**Variable meaning**
MPI	Numeric	Between 0 and 1
Age	Numeric	Between 16 and 59
Gender	Categorical	Male = 1; female = 0
Hukou status	Categorical	Non-agricultural = 1; agricultural = 0
Disability categories	Numeric	Between 1 and 7
Disability grades	Numeric	Between 1 and 4
Marital status	Categorical	With spouse = 1; without spouse = 0
Social assistance	Categorical	Get = 1; not get = 0
Welfare subsidies	Categorical	Get = 1; not get = 0
Rehabilitation services	Categorical	Get = 1; not get = 0
Barrier-free reconstruction	Categorical	Get = 1; not get = 0
Employment opportunity	Categorical	Yes = 1; no = 0
Vocational skill	Numeric	Between 0 and 10
Internet employment	Categorical	Yes = 1; no = 0

Persons with disabilities aged 16–59 who are not incapacitated and in need of employment services are mainly provided with employment services. The samples of studying in school, retirement and no employment intention were deleted in this part, and finally 57,510 people in 2018 were selected and the information in 2019 was tracked (total sample of 115,020 in two periods). The descriptive statistics of each variable are shown in Table 6. It can be seen that the MPI of persons with disabilities who received employment services was 0.323, and that of persons with disabilities who did not receive employment services was 0.344. The MPI of persons with disabilities who received employment services was significantly lower. In addition, the mean values of employment opportunities, employment skills and Internet employment for persons with disabilities who received employment services were higher than those who did not receive employment services.

**Table 6:** Descriptive statistics of variables.

**Variable**	**All**				**Employment services**		**No employment services**	
	**(*****n*** = **115,020)**				**(*****n*** = **20,443)**		**(*****n*** = **94,577)**	
	**Mean**	**SD**	**Min**	**Max**	**Mean**	**SD**	**Mean**	**SD**
MPI	0.340	0.159	0	1	0.323	0.161	0.344	0.158
Age	45.918	9.203	16	59	46.122	8.997	45.874	9.246
Gender	0.682	0.466	0	1	0.705	0.456	0.677	0.468
Hukou status	0.263	0.440	0	1	0.161	0.368	0.285	0.452
Disability categories	4.572	1.366	1	7	4.580	1.324	4.571	1.375
Disability grades	3.047	0.941	1	4	3.075	0.940	3.041	0.941
Marital status	0.751	0.433	0	1	0.760	0.427	0.749	0.434
Social assistance	0.472	0.499	0	1	0.574	0.495	0.450	0.497
Welfare subsidies	0.352	0.478	0	1	0.432	0.495	0.335	0.472
Rehabilitation services	0.218	0.413	0	1	0.334	0.472	0.192	0.394
Barrier-free reconstruction	0.021	0.143	0	1	0.057	0.231	0.013	0.113
Employment opportunity	0.620	0.485	0	1	0.762	0.426	0.589	0.492
Vocational skill	0.406	0.541	0	18	0.708	0.590	0.341	0.507
Internet employment	0.012	0.108	0	1	0.022	0.145	0.010	0.098

#### 3.2.2. OLS regression analysis

The OLS method in this part is used to preliminarily investigate the relationship between employment services and multidimensional poverty of persons with disabilities, and the results are shown in Table 7. As China implements various support policies for the disabled, the multidimensional poverty of the disabled will not only be impacted by employment services, but also by other social support. In order to enhance the robustness of the model, the personal characteristics and social support are gradually incorporated into the model. The results of models (1), (3) and (5) show that the correlation coefficients between employment services and the MPI of persons with disabilities are −0.028, −0.012 and −0.020, respectively, which are significant at the level of 0.1%, indicating that employment services can significantly reduce the MPI of persons with disabilities. After adding social support indicators, the results of models (2), (4) and (6) show that the correlation coefficients between employment services and the MPI of persons with disabilities are −0.034, −0.018 and −0.024, respectively, which are also significant at the level of 0.1%, indicating that employment services can still significantly reduce the MPI of persons with disabilities after controlling other policy shocks. The empirical analysis proves that China's current employment services have played a greater role in solving the poverty problem of the disabled in the process of poverty alleviation, and the effect of employment intervention measures is not as negative as expected.

**Table 7:** Effect of employment services on MPI: OLS estimate result.

**Variable**	**2018**		**2019**		**All**	
	**(1)**	**(2)**	**(3)**	**(4)**	**(5)**	**(6)**
Employment service	−0.028^***^ (0.002)	−0.034^***^ (0.002)	−0.012^***^ (0.002)	−0.018^***^ (0.002)	−0.020^***^ (0.001)	−0.024^***^ (0.001)
Age	0.002^***^ (0.000)	0.002^***^ (0.000)	0.002^***^ (0.000)	0.001^***^ (0.000)	0.001^***^ (0.000)	0.001^***^ (0.000)
Gender	−0.021^***^ (0.001)	−0.021^***^ (0.001)	−0.024^***^ (0.001)	−0.024^***^ (0.001)	−0.022^***^ (0.001)	−0.022^***^ (0.001)
Hukou status	−0.016^***^ (0.001)	−0.018^***^ (0.001)	−0.004^*^ (0.001)	−0.007^***^ (0.001)	−0.010^***^ (0.001)	−0.013^***^ (0.001)
Disability categories	0.006^***^ (0.000)	0.005^***^ (0.000)	0.005^***^ (0.000)	0.004^***^ (0.000)	0.005^***^ (0.000)	0.005^***^ (0.000)
Disability grades	−0.031^***^ (0.001)	−0.023^***^ (0.001)	−0.032^***^ (0.001)	−0.023^***^ (0.001)	−0.031^***^ (0.000)	−0.023^***^ (0.001)
Marital status	−0.066^***^ (0.002)	−0.061^***^ (0.002)	−0.066^***^ (0.001)	−0.059^***^ (0.001)	−0.065^***^ (0.001)	−0.059^***^ (0.001)
Social assistance		0.027^***^ (0.001)		0.038^***^ (0.001)		0.034^***^ (0.001)
Welfare subsidy		0.026^***^ (0.002)		0.022^***^ (0.002)		0.023^***^ (0.001)
Rehabilitation service		−0.006^**^ (0.002)		−0.003^*^ (0.001)		−0.020^***^ (0.001)
Barrier-free reconstruction		0.020^***^ (0.005)		−0.032^***^ (0.004)		−0.015^***^ (0.003)
Constant	0.425^***^ (0.004)	0.385^***^ (0.004)	0.392^***^ (0.004)	0.346^***^ (0.004)	0.412^***^ (0.003)	0.372^***^ (0.003)
*N*	57,510	57,510	57,510	57,510	115,020	115,020
Pseudo R2	0.077	0.093	0.081	0.103	0.075	0.096
F	682.105	535.262	722.540	603.083	1,340.193	1,115.182

* *p* < 0.01,

** *p* < 0.005,

*** *p* < 0.001; standard errors are given in brackets.

#### 3.2.3. PSM-DID analysis

In order to more accurately investigate the poverty reduction effect of employment services on the multidimensional poverty of the disabled and solve the endogenous problems such as sample selection bias and missing variables, PSM-DID method is used for analysis in this part. The treatment group is the disabled who did not receive employment services in 2018 and received employment services in 2019, and the control group is the disabled who did not receive employment services in 2018 and 2019. There are 6,482 samples in the treatment group and 40,626 samples in the control group.

The effect of employment services on MPI and different poverty dimensions is shown in Table 8. The DID value is the result of our main concern and represents the effect of employment services on the multidimensional poverty of persons with disabilities. The DID value of the MPI is −0.015 (*p* = 0.000), which means that the MPI of persons with disabilities has decreased by 0.015 units after receiving employment services, indicating that employment services have a significant effect on improving the multidimensional poverty of persons with disabilities. In order to further analyze the role of employment services in improving each poverty dimension, this paper uses the same method to study the five poverty dimensions of the disabled respectively. The results show that the DID values of the poverty index in economy, education, insurance and social participation are −0.002 (*p* = 0.008), −0.003 (*p* = 0.008), −0.005 (*p* = 0.000) and −0.008 (*p* = 0.000), respectively, which means that the poverty index in the four dimensions decreases by 0.002 units, 0.003 units, 0.005 units and 0.008 units respectively after the disabled obtain employment services. It can be seen that employment services have a positive impact on many areas of life for the disabled. As pointed out by relevant scholars, assessing changes attributable to vocational interventions should focus on the impact of improvement of life skills through vocational interventions on multiple areas of quality of life (17). It is worth noting that employment services play a greater role in improving the poverty of education, insurance and social participation than in economic poverty, which to some extent indicates that China provides a minimum guarantee in ensuring that the disabled and their families have a decent income (12).

**Table 8:** Effect of employment services on MPI and different poverty dimensions.

		**MPI**	**Economy**	**Health**	**Education**	**Insurance**	**Social participation**
		**(1)**	**(2)**	**(3)**	**(4)**	**(5)**	**(6)**
Before	Control	0.366	0.027	0.005	0.089	0.101	0.170
	Treated	0.370	0.033	0.005	0.088	0.100	0.171
	Diff (T-C)	0.005^***^	0.004^***^	0.000	−0.001	−0.001	0.001
After	Control	0.329	0.018	0.003	0.085	0.088	0.165
	Treated	0.318	0.021	0.005	0.081	0.082	0.156
	Diff (T-C)	−0.011^***^	0.002^**^	0.003^***^	−0.004^***^	−0.006^***^	−0.009^***^
DID		−0.015^***^	−0.002^**^	0.003^***^	−0.003^**^	−0.005^***^	−0.008^***^
t		7.41	2.67	6.95	2.64	4.94	13.9
p		0.000	0.008	0.000	0.008	0.000	0.000
Control variable		Yes	Yes	Yes	Yes	Yes	Yes
*N*		94,216	94,216	94,216	94,216	94,216	94,216

** *p* < 0.005,

*** *p* < 0.001.

The effect of employment services on MPI of different disability categories is shown in Table 9. DID value is the result of our main concern and represents the change of MPI of different categories of disabled people after receiving employment services. The DID results of models (1) – (7) are −0.026 (*p* = 0.000), −0.018 (*p* = 0.003), −0.011 (*p* = 0.007), −0.011 (*p* = 0.000), −0.023 (*p* = 0.001), −0.015 (*p* = 0.049), −0.014 (*p* = 0.213), respectively. It can be seen that, compared with other types of disabilities, employment services have the greatest effect on poverty reduction for persons with visual disabilities, which may be related to China's strong support for the development of blind massage industry and encouraging the scale and branding of blind massage during the 13th Five-year period. It is worth noting that, the DID results of persons with psychiatric disabilities or multiple disabilities are not significant, which means that employment services have little effect on poverty reduction of these two types of disabled people. In the post-poverty alleviation era, China needs to adjust the employment service measures for these two categories of disabled people.

**Table 9:** Effect of employment services on MPI of different disability categories.

		**Visual disability**	**Hearing disability**	**Speech disability**	**Physical disability**	**Intellectual disability**	**Psychiatric disability**	**Multiple disability**
		**(1)**	**(2)**	**(3)**	**(4)**	**(5)**	**(6)**	**(7)**
Before	Control	0.353	0.378	0.413	0.344	0.477	0.406	0.447
	Treated	0.375	0.388	0.416	0.349	0.483	0.4	0.463
	Diff (T-C)	0.022^***^	0.010^**^	0.003	0.005^***^	0.005	−0.006	0.016^*^
After	Control	0.323	0.335	0.381	0.303	0.451	0.38	0.413
	Treated	0.319	0.327	0.373	0.296	0.433	0.359	0.414
	Diff (T-C)	−0.004	−0.008^*^	−0.008^*^	−0.006^***^	−0.018^***^	−0.022^***^	0.002
DID		−0.026^***^	−0.018^**^	−0.011^*^	−0.011^***^	−0.023^***^	−0.015	−0.014
t		3.94	2.94	6.95	4.44	3.35	1.97	1.25
p		0.000	0.003	0.007	0.000	0.001	0.049	0.213
Control variable		Yes	Yes	Yes	Yes	Yes	Yes	Yes
*N*		9,131	10,116	1,751	58,031	6,373	6,335	2,294

* *p* < 0.01,

** *p* < 0.005,

*** *p* < 0.001.

#### 3.2.4. Mediation analysis

From the above analysis, we can see that employment services can effectively improve poverty in economy, education, insurance and social participation, and promote the reduction of MPI. The starting point of employment services is to improve employment skills, promote employment and enrich employment forms, and the quality of life for persons with disabilities is improved by achieving these vocational outcomes. Then whether employment services will improve multidimensional poverty by achieving vocational outcomes, and the mediation effects of employment opportunities, vocational skills and Internet employment are verified in this part.

The mediation effect results of employment opportunities are shown in Table 10. The correlation coefficient between employment services and employment opportunities of persons with disabilities in model (2) is 0.118, which is significantly at the level of 0.1%, indicating that employment opportunities of the disabled increases by 0.118 units after receiving employment services. Employment services and employment opportunities are introduced into model (3) at the same time. The correlation coefficient between employment services and the MPI in model (3) is −0.010, which is lower in absolute value compared to the coefficient in model (1), indicating that employment services can improve multidimensional poverty by promoting the employment of persons with disabilities. The results show that employment opportunities have a partial mediation effect in the relationship between employment services and multidimensional poverty.

**Table 10:** Results of the mediation effect test of employment opportunities.

**Variable**	**MPI**	**Employment opportunity**	**MPI**
	**(1)**	**(2)**	**(3)**
Employment service	−0.020^***^ (0.002)	0.118^***^ (0.005)	−0.010^***^ (0.002)
Employment opportunity			−0.075^***^ (0.001)
Control variable	Yes	Yes	Yes
Individual fixed effects	Yes	Yes	Yes
Time fixed effects	Yes	Yes	Yes
Constant	0.382^***^ (0.022)	0.480^***^ (0.068)	0.417^***^ (0.021)
Pseudo R2	0.103	0.068	0.151

*** *p* < 0.001; standard errors are given in brackets.

The mediation effect results of vocational skills are shown in Table 11. The correlation coefficient between employment services and vocational skills of persons with disabilities in model (2) is 0.329, which is significantly at the level of 0.1%, indicating that the vocational skills of persons with disabilities increase by 0.329 units after obtaining employment services. Employment services and vocational skills are introduced into model (3) at the same time. The correlation coefficient between employment services and the MPI in model (3) is −0.010, which is lower in absolute value compared to the coefficient in model (1), indicating that employment services can improve multidimensional poverty by improving the vocational skills of persons with disabilities. The results show that vocational skills have a partial mediation effect in the relationship between employment services and multidimensional poverty.

**Table 11:** Results of the mediation effect test of vocational skills.

**Variable**	**MPI**	**Vocational skill**	**MPI**
	**(1)**	**(2)**	**(3)**
Employment service	−0.020^***^ (0.002)	0.329^***^ (0.006)	−0.010^***^ (0.002)
Vocational skill			−0.031^***^ (0.001)
Control variable	Yes	Yes	Yes
Individual fixed effects	Yes	Yes	Yes
Time fixed effects	Yes	Yes	Yes
Constant	0.382^***^ (0.022)	0.383^***^ (0.078)	0.394^***^ (0.022)
Pseudo R2	0.103	0.089	0.115

*** *p* < 0.001; standard errors are given in brackets.

The mediation effect results of Internet employment are shown in Table 12. The correlation coefficient between employment services and Internet employment of persons with disabilities in model (2) is 0.011, which is significantly at the level of 0.1%, indicating that the probability of Internet employment of persons with disabilities increase by 0.011 units after receiving employment services. Employment services and Internet employment are introduced into model (3) at the same time. The correlation coefficient between employment services and the MPI in model (3) is −0.019, which is slightly lower in absolute value compared to the coefficient in model (1), indicating that employment service can improve multidimensional poverty by promoting Internet employment for persons with disabilities. Internet employment has a mediation effect, but this effect is very limited.

**Table 12:** Results of the mediation effect test of Internet employment.

**Variable**	**MPI**	**Internet employment**	**MPI**
	**(1)**	**(2)**	**(3)**
Employment service	−0.020^***^ (0.002)	0.011^***^ (0.001)	−0.019^***^ (0.002)
Internet employment			−0.107^***^ (0.006)
Control variable	Yes	Yes	Yes
Individual fixed effects	Yes	Yes	Yes
Time fixed effects	Yes	Yes	Yes
Constant	0.382^***^ (0.022)	0.045^***^ (0.016)	0.389^***^ (0.022)
Pseudo R2	0.103	0.007	0.110

*** *p* < 0.001; standard errors are given in brackets.

## 4. Discussion

This paper uses the data of the disabled in Jilin Province, China to measure the levels of multidimensional poverty of the disabled. The results show that among persons with disabilities aged 16–59, about 90% are deprived in at least one dimension, and about 30% are in a state of severe multidimensional poverty until 2019. One study showed that 41.1% of the population with disabilities in Peru suffer deprivations in at least three out of the eight dimensions (32). Although scholars choose different indicators for multidimensional poverty in different countries and the results cannot be directly compared, it can at least prove that serious multidimensional poverty is widespread among people with disabilities, which is a common phenomenon in many countries, especially low- and middle-income countries (6, 8, 34). In addition, the degree of poverty in different dimensions is different. The results on contribution of economy, health and insurance are relatively low, indicating that the basic livelihood of persons with disabilities has been guaranteed to a certain extent. The results on contribution of education and social participation are relatively high, indicating that the disabled are obviously insufficient in their ability to learn and integrate into society. This phenomenon is not only found in China, but also in most countries (6, 8). Persons with disabilities are underrepresented in all forms of cultural life and suffer from severe social exclusion (41). Moreover, it is more difficult to meet the needs of the disabled in education services, rehabilitation programs, social services and other development than to meet their basic living needs and provide income security benefits.

While multidimensional poverty is prevalent among persons with disabilities, there has been significant progress in economy, social and community participation over the past 25 years. In part, these advancements are due to improving policy frameworks and laws, occupational and vocational rehabilitation strategies, and corporate practices in support of a more diverse and inclusive workforce (42). The results of this study show that the MPI of the disabled decreased in 2019 compared with that in 2018, which indicates that the multidimensional poverty degree of the disabled has been reduced and the overall trend of multidimensional poverty of the disabled in China is improving. However, different poverty dimensions show different development situations. The results on contribution of economy, health and insurance decreased significantly in 2019 compared with that in 2018. Because these three dimensions are closely related to the basic life of the disabled, the decline in the contributions of these three dimensions represents that the basic living security of the disabled in China has achieved positive results in recent years. During the 13th Five-year period, China took the cause of the disabled as an important part of ensuring people's livelihood, continuously improved the medical security system for the disabled and made a series of welfare reforms, which greatly improved the basic living standards of the disabled. In addition, the contributions of education and social participation are still high, indicating that persons with disabilities are more seriously deprived in the dimensions of education and social participation. Education and social participation are higher-level needs of persons with disabilities. Due to physical defects, disabled people face many difficulties in these two dimensions. In order to ensure that the disabled have equal rights to education and participate in social life, China has taken relevant measures to intervene. In 2012, China promulgated the Regulations on the Construction of Barrier Free Environment (State Council 2012–622), and in 2017, China issued the revised Regulations on Education for Persons with Disabilities (State Council 2017–674). However, it is not so easy to implement these regulations, which leads to slow progress in education and social participation of the disabled. Therefore, in the post-poverty era, China needs to pay attention to the development needs of disabled people on the premise of ensuring their survival.

In order to improve the wellbeing of persons with disabilities, the disability policy of the welfare state has generally undergone a convergence transition from income maintenance to employment incentives (43, 44). The Chinese government has always attached great importance to encouraging and helping disabled persons to find employment through various means, but there is a lack of research on the effects of employment policies for disabled persons in China. First, the results of this study show that the MPI has decreased significantly after persons with disabilities receiving employment services, indicating that employment services have played a great role in reducing poverty. As studied in other countries, employment intervention and occupational rehabilitation not only improve vocational outcomes, but also improve education, community integration, quality of life and other areas of life (15, 45). Second, after the disabled receive employment services, the poverty dimensions of economy, education, insurance and social participation have been improved to varying degrees, and the poverty dimensions of education, insurance, and social participation are improved to a stronger degree than economy. The possible reason is that employment services generate income by promoting employment, but the employment quality of the disabled is not high and the promotion space is small. Moreover, that the traditional mindset is that it is acceptable for persons with disabilities to be relegated to lower-paying, less-valued jobs that are separate from mainstream employment opportunities, so the degree of employment services to improve the economic ability of persons with disabilities is limited. This also reflects, to some extent, that Chinese government legislation asserts the protection of the rights of persons with disabilities but provides minimal direction in ensuring that the disabled and their families have a decent income (12). Finally, the research results of different categories of people with disabilities showed that employment services did not significantly improve multidimensional poverty for people with psychiatric or multiple disabilities. This is inconsistent with previous studies (15–18), suggesting that the effect of employment services provided to people with psychiatric or multiple disabilities in China is extremely limited from the perspective of multidimensional poverty compared to other countries. In the post-poverty alleviation era, China needs to adjust the employment service measures for these two categories of disabled people.

This study further verifies the mediation mechanism of the relationship between employment services and multidimensional poverty of the disabled. The results show that employment opportunities, vocational skills and Internet employment have a partial mediation effect in this relationship. That is to say, employment services can improve multidimensional poverty of persons with disabilities by obtaining some vocational outcomes. This is similar to related research findings in other countries. Some studies pointed out that receiving counseling/guidance, on-the-job training, job search assistance, on-the-job supports, information/referral services, maintenance, and supported employment were positive predictors of competitive employment (45, 46). These services may have alleviated some of the barriers to employment for persons with disabilities including inadequate training, lack of information, unavailability of jobs, and negative employer perceptions (47). At the same time, employment can enhance problem-solving confidence, social support, sense of community and network ties of persons with disabilities, and has long been associated with quality of life and wellbeing (48, 49). In addition, it should be noted that the effects of the three mediation variables are different. Compared with employment opportunities and vocational skills, the mediation effect of Internet employment is lower, which means that employment services have a limited effect on promoting Internet employment. On the one hand, disabled people are often excluded from digital life due to barriers like high cost, poor design of devices and software, and the lack of relevant knowledge and skills (50, 51). It is difficult for the disabled to obtain employment through the Internet. On the other hand, China has increased its support for the new employment form of “Internet Plus” in the Protocols of the Promotion of Disability Employment in the 13th Five-year period, but the relevant policies are still in their infancy, and the obstacles in their implementation and promotion lead to limited effect of employment services in promoting Internet employment. Internet employment has a positive and effective role in the lives of persons with disabilities, and it is necessary to continuously improve the relevant policy intervention system.

We have obtained some meaningful conclusions by evaluating the effect of employment services on multidimensional poverty, but this study also has some limitations. First, due to data limitations, eight indicators are used to measure multidimensional poverty of persons with disabilities in the current analysis, however, many other indicators (such as per capita income, nutrition, self-rated health, living standards, etc.) could be used to measure multidimensional poverty of the disabled more deeply. Second, employment service is operated as a comprehensive indicator in this paper, and the research on its poverty reduction effect focuses on whether persons with disabilities have received a certain service. However, the type and intensity of employment services received by persons with disabilities are equally critical to outcomes, and this section deserves further study.

## Data availability statement

The original contributions presented in the study are included in the article/Supplementary material, further inquiries can be directed to the corresponding author.

## Ethics statement

Ethical review and approval was not required for the study on human participants in accordance with the local legislation and institutional requirements. Written informed consent from the participants' legal guardian/next of kin was not required to participate in this study in accordance with the national legislation and the institutional requirements.

## Author contributions

XW and JG conceived the idea. JG and HL processed the data and drafted the manuscript. XW, JG, and HL revised and polished this article. All authors contributed to the article and agree to submit this version.
