# Supplementary material for: Multidimensional poverty of persons with disabilities in China: An analysis of poverty reduction effect of employment services
Source: Front Public Health. 2023 Feb 10;11:1093978. doi: 10.3389/fpubh.2023.1093978 (PMC9950746; doi:10.3389/fpubh.2023.1093978)
Supplement: Supplementary file 1 [file Data_Sheet_1.pdf]

Dynamic Update of Basic Service Status and Demand Information of the Disabled in Jilin Province

Registration Form for the Disabled

Table No.: JL745
Prepared by: Jilin Disabled Persons' Federation
Approved by: Statistic Bureau of Jilin
Approval No.: JTSZ [2019] No. 4
Expiry date: July 2021

________ **County (district, city)** _______ **Township (town, street)**
________ **Village (community)**

**R1. Name**		**R2. ID Number**	
**B1. Name of householder**		**B2. ID number of household**	
**R3. Hukou status**	1. Agricultural hukou<br>2. Non-agricultural hukou	**R4. Marital status** (★Filled in at the age of 20 and above)	1. Unmarried 2. Married with spouse 3. Divorced 4. Widowed
**D1. Family demographics** (★Choose all those apply)		1. The old and the disabled 2. Multiple disabled persons in one household<br>3. Integration of disability and disease 4. Other	
**R5. Contact name** (★Filled in by the mentally disabled, the mentally disabled and the disabled aged 17 and below)		**R6. Contact number of disabled person or contact person**	Telephone: Mobile phone:
**R7. Whether you live in a senior (nursing) home, a welfare home, an Invalids Home, etc.**	1.Yes (★Fill in only three parts: education, social security, and basic medical care and rehabilitation)<br>2.No		

Economy and Housing

★Non-agricultural hukou	**R8. Family income status**	1. Below the minimum living standard<br>2. Below the low-income standard or the minimum living standard margin 3. Other
	**R9. Family housing status**	1. Own property house 2. Housing security policies (low rent house or public rental house, etc.)<br>3. Rental house 4. Borrowed or no fixed domicile
★ Agricultural hukou	**R10. Poverty and file status**	1. People not out of poverty registered by the state 2. Other poor people 3. Other
	**R11. Family housing status**	1. In good condition 2. Identified as dangerous house<br>3. Dangerous buildings not identified 4. Rental house<br>5. Borrowed or no fixed residence

Education

**R12. Literate** (★Filled in at the age of 15 and above)	1. Yes (Urban residents can read more than 2000 words, farmers can read more than 1500 words, or can read popular newspapers or write notes)<br>2. No
**R13. Education** (★Filled in at the age of 15 and above)	1. No formal education 2. Elementary school 3. Middle school 4. High school (including vocational school) 5. Associate degree 6. Bachelor's degree 7. Postgraduate degree
**R14. Current school** (★Filled in by disabled children and students in kindergarten and school))	**General educational institutions:** 1. Preschool 2. Elementary school 3. Middle school 4. High school (including general education and vocational education) 5. Associate degree 6. Bachelor's degree or above<br>**Special education institutions:** 7. Preschool 8. Elementary school 9. Middle school 10. High school (including general education and vocational education) 11. Associate degree 12. Bachelor's degree or above
**D2. Whether to enjoy the door-to-door education service (filled in by disabled children aged 6-14)**	1. Yes 2. No

★Filled in by disabled children aged 6-14 who are not enrolled in school	**R15. Main reasons for not attending school**	1. Severe disability 2. Family financial difficulties<br>3. No school admission 4. Inconvenient transportation<br>5. Postponing the school age 6. Parents have no intention
	**D3. Whether there is a demand for door-to-door education service**	1. Yes 2. No
**Poverty Alleviation through Employment (filled in at the age of 16-59)**		
**R16. Whether employed**		1. Yes 2. No
**R17. Employment forms for persons with disabilities** (skip to R20)		1. Proportionate employment 2. Concentrative employment<br>3. Individual employment (including independent entrepreneurship) 4. Public welfare employment<br>5. Auxiliary employment 6. Rural planting and breeding 7. Flexible employment
**R18. Main sources of livelihood of the unemployed disabled**		1. Pension 2. Property income 3. Social assistance and social welfare<br>4. Support from family members 5. Other
**R19. Main Reasons for Unemployment**		1. Studying at school (skip to R25) 2. Retirement (skip to R22)<br>3. No employment intention (skip to R22) 4. No employment skills<br>5. Loss of labor ability (skip to R22) 6. Agricultural land expropriated 7. Other
**D4. Your current employment intention** (Filled in by the unemployed disabled; items 1-5 can be selected more than one)		1. Proportionate employment 2. Concentrative employment<br>3. Individual employment (including independent entrepreneurship) 4. Public welfare employment<br>5. Auxiliary employment 6. Rural planting and breeding 7. Flexible employment
**R20. Employment and poverty alleviation assistance received in the year** (★Items 1-5 can be selected more than one)		1. Vocational skills training 2. Career introduction 3. Rural practical technical training<br>4. Funding and credit support 5. Other assistance 6. None
**R21. Current employment demand** (Choose all those apply)	1. Vocational skills training (Choose all those apply)	① Home appliance repair ② Beauty salon ③ Handmade<br>④ Computer operation and maintenance<br>⑤ Car beauty ⑥ Blind massage ⑦ Motor vehicle repair ⑧ E-commerce<br>⑨ Creative design ⑩ Enterprise management ⑪ Plumbing ⑫ Accounting<br>⑬ Pastry making ⑭ Hydroelectric welding ⑮ Health and medical care<br>⑯ Sheet metal ⑰ Rural planting and breeding
	2. Career introduction (Choose all those apply)	① Vocational counseling ② Job registration<br>③ Vocational guidance ④ Intermediary services
	3. Rural practical technical training (Choose all those apply)	① Planting technology ② Breeding technology<br>③ Processing of agricultural products
	4. Funding and credit support (Choose all those apply)	① Fund subsidy ② Credit guarantee<br>③ Interest reduction ④ Equipment and site subsidy
	5. Other assistance (Choose all those apply)	① Employment and unemployment registration ② Psychological counseling<br>③ Occupational skill identification ④ Employment Training<br>⑤ Other
	6. None	
**D5. What you have mastered employment skills** (★Items 1-18 can be selected more than one)		1. Home appliance repair 2. Beauty salon 3. Handmade 4. Computer operation and maintenance<br>5. Car beauty 6. Blind massage 7. Motor vehicle repair 8. E-commerce<br>9. Creative design 10. Enterprise management 11. Plumbing 12. Accounting<br>13. Pastry making 14. Hydroelectric welding 15. Health and medical care 16. Sheet metal<br>17. Rural planting and breeding 18. Other<br>19. None (if this item is selected, other items cannot be selected)
**Social Security**		
**R22. Whether to participate in social insurance for employees** (★Filled in at the age of 16 and above; items 1-3 can be selected more than one)		1. Pension insurance 2. Medical insurance<br>3. Other insurances (unemployment insurance, employment injury insurance and birth insurance)<br>4. None

**R23. Whether to participate in pension insurance for urban and rural residents**<br>(★ Filled in at the age of 16 and above)	1. Yes<br>2. No	**R24. Is there any pension insurance payment subsidy for urban and rural residents**<br>（★Filled in at the age of 16-59）	1. Yes<br>2. No
**R25. Whether to participate in medical insurance (Urban residents/new rural cooperative medical system)**	1. Yes<br>2. No（skip to R27）	**R26. Is there any medical insurance payment subsidy**	1. Yes<br>2. No
**R27. Social assistance and housing improvement in the year**<br>(★Items 1-5 can be selected more than one)	1. Minimum living security 2. Assistance and support for people in special need<br>3. Medical assistance 4. Other assistance (education assistance, housing assistance, employment assistance and other temporary assistance)<br>5. Enjoy the rural dilapidated house reconstruction policy of the housing construction department (★ Only for agricultural hukou) 6. None		
**R28. Social welfare subsidies**<br>(★Items 1-3 can be selected more than one)	1. Living allowance for the disabled with difficulties<br>2. Nursing allowance for the severely disabled 3. Other welfare subsidies 4. None		
**Foster care services** (★ Filled in by persons with intellectual, mental and severe physical disabilities aged 16-59) — **R29. Is there childcare service**	1. Yes (skip to R31) 2. No		
**R30. Current demand for childcare services**	1. Home service 2. Day care 3. Boarding care 4. None		
**Elderly care services** (★ Filled in by the disabled over 60 years old) — **D6. Is there elderly care services**	1. Yes (skip to R31) 2. No		
**D7. Current demand for elderly care services**	1. Home based elderly care service 2. Institutional elderly care service<br>3. Community day-care 4. None		
**Basic Medical Care and Rehabilitation**			
**Basic medical care** — **R31. Whether an individual or family has signed a family doctor service agreement (registered residence + city / state)**	1. Yes 2. No		
**R32. Other diseases besides disability in the past two weeks**	1. Yes<br>2. No (skip to R35)	**R33. Have you seen or treated in the past two weeks**	1. Yes (skip to R35)<br>2. No
**R34. Reasons for not seeing a doctor or treatment**<br>(★Choose all those apply)	1. Mild illness 2. Economic difficulties 3. Trouble in seeing a doctor<br>4. No time 5. Other		
**R35. Have you received or used the following services in the year**<br>(★Items 1-5 can be selected more than one)	1. Operation 2. Medicine 3. Functional training<br>4. Auxiliary equipment 5. Nursing (supporting services) 6. None		
**R36. Reasons for not receiving rehabilitation services**<br>(★Fill in this item if R35 selects 6, and skip this item for others; choose all those apply)	1. Lack of knowledge and information 2. Economic difficulties<br>3. Inconvenient traffic 4. Other		
**D8. Whether you have received or used the following supporting services in the year (**★Items 1-7 can be selected more than one**)**	1. Psychological counseling 2. Parental rehabilitation guidance 3. Rehabilitation counseling<br>4. Rehabilitation referral. 5. Daytime care for severely disabled persons<br>6. Work (entertainment) therapy, agricultural therapy 7. Occupational rehabilitation and other services 8. Not obtained (if this item is selected, other items cannot be selected)		
**R37. Do** — 1. Operation (Choose all those apply)	① Cataract vision restoration surgery ② Limb correction surgery ③ Cochlear implantation surgery		

you still need the following services for your disability (★ Choose all those apply)	2. Drugs (Choose all those apply)	① Psychotic medication
	3. Function training (Choose all those apply)	Vision: ① Directional walking and adaptive training ② Low vision visual function training<br>Hearing language: ③ Hearing speech ability training ④ Sign language guidance<br>Limbs: ⑤ Sports and adaptive training<br>Intelligence: ⑥ Cognitive and adaptive training<br>Spirit: ⑦ Communication and adaptation training for autistic children<br>⑧ Occupational therapy training for mental disorders<br>⑨ Occupational therapy, agricultural therapy ⑩ Entertainment therapy and physical therapy
	4. Auxiliary equipment (Choose all those apply)	Vision: ① Visual aids ② Blind staff<br>Hearing: ③ Hearing aids ④ Cochlear implants<br>Speech: ⑤ Conversation communication tools<br>Limbs: ⑥ Wheelchairs ⑦ Prosthetics ⑧ Orthotics ⑨ Walking aids ⑩ Self-help appliances<br>Intelligence: ⑪Intelligence toys
	5. Care (Choose all those apply)	① Living care ② Personal hygiene assistance ③ Psychological assistance and support<br>④ Basic treatment ⑤ Health guidance
	6. None	

Barrier Free Environment Construction and Legal Service

**R38. Has there been a barrier-free reconstruction this year**	1. Yes 2. No
**R39. What are your current needs for barrier-free reconstruction** (★Items 1-10 can be selected more than one)	1. Sloping and handrails at the door 2. Door reconstruction 3. Toilet reconstruction<br>4. Kitchen reconstruction 5. Flashing doorbell and visual doorbell (for the deaf)<br>6. Gas leakage alarm sounding device (for the blind)<br>7. Online screen reading software (for the blind) 8. Installing shower<br>9. Yard leveling 10. Other 11. None
**D9. What are your current public accessibility needs** (★Items 1-8 can be selected more than one)	1. Barrier-free facilities in public bathrooms 2. Barrier-free facilities in public bathrooms<br>3. Audio prompt device for crossing the street 4. Barrier-free websites<br>5. TV news sign language subtitles 6. Barrier-free parking spaces for motor vehicles<br>7. Blind track and curb ramp 8. Other<br>9. None (if this item is selected, other items cannot be selected)
**D10. Have you received legal services this year**	1. Yes 2. No
**D11. What legal services do you need at present** (★ Items 1-5 can be selected more than one)	1. Consultation 2. Proxy 3. Litigation agency<br>4. Popularization of legal knowledge 5. Other 6. None

Culture and Sports (6 years old and above)

**D12. Personal literary and artistic expertise** (★Items 1-8 can be selected more than one)	1. Music 2. Dance 3. Performance 4. Calligraphy and painting<br>5. Literary creation 6. Photography 7. Handmade crafts 8. Other 9. None
**D13. Personal sports expertise** (★Items 1-11 can be selected more than one)	1. Track and field 2. Basketball 3. Volleyball 4. Football 5. Table tennis<br>6. Badminton 7. Weightlifting 8. Bicycle 9. Ice and snow events<br>10. Shooting (including archery) 11. Other 12. None
**D14. Do you use the Internet (Including using intelligent phone)**	1. Yes (_____ hours a day online) 2. No (skip to R40)
**D15. Your Internet Access** (★Choose all those apply)	1. PC Internet Access 2. Mobile Internet Access
**D16. Your main use of the Internet** (★Items 1-6 can be selected more than one)	1. Access to information 2. Online learning 3. Online employment and entrepreneurship<br>4. Online shopping 5. Communication 6. Online entertainment

**R40. Do you often participate in cultural and sports activities organized by the village (community) this year**	1. Yes (skip to D17) 2. No
**R41. Reasons for not regularly participating in cultural and sports activities organized by the village (community)** (★Choose all those apply)	1. No suitable activities 2. No suitable places and facilities<br>3. No one organizes guidance 4. Other
**D17. Current stylistic requirements** (★Items 1-4 can be selected more than one)	1. Hold cultural and sports activities<br>2. Participate in the guidance and training of arts and sports majors<br>3. Provide fitness training 4. Configure recreational and sports equipment 5. None

Declared by: Information collector: Date of filling:
